# Tax increases as an incentive to quit smoking: is thinking about quitting due to a tobacco tax increase associated with post-tax increase smoking cessation?

**DOI:** 10.1186/s12889-024-19530-6

**Published:** 2024-07-25

**Authors:** Cloé Geboers, Math J. J. M. Candel, Gera E. Nagelhout, Bas van den Putte, Marc C. Willemsen

**Affiliations:** 1 Department of Health Promotion (CAPHRI), Maastricht University, P. Debyeplein 1, 6221 HA Maastricht, The Netherlands; 2 The Netherlands Expertise Centre for Tobacco Control, Trimbos Institute, Utrecht, the Netherlands; 3 Department of Methodology and Statistics (CAPHRI), Maastricht University, Maastricht, the Netherlands; 4 Centre of Expertise Perspective in Health, Avans University of Applied Sciences, Breda, the Netherlands; 5 Department of Communication (ASCoR), University of Amsterdam, Amsterdam, The Netherlands

**Keywords:** Tobacco, Smoking cessation, Taxation, Price, Policy, Longitudinal research

## Abstract

**Background:**

The cost of tobacco is one of the most reported reasons to quit smoking. The Netherlands increased tobacco taxes twice in the span of nine months: a €1 increase per pack in April 2020, and a €0.12 increase per pack in January 2021. This study examines to what extent people report to think about quitting due to the upcoming tax increase(s), as well as how it relates to their age, income or educational level. Additionally, we examined whether thinking about quitting was associated with quit intention and quit behaviour, and whether these associations were different for the two tax increases.

**Methods:**

Longitudinal data from the International Tobacco Control (ITC) Netherlands Surveys, Cohort 2 were used (*N* = 5919 observations; wave 1 (February – March 2020): *n* = 2051; wave 2 (September – November 2020): *n* = 1919; wave 3 (June – July 2021): *n* = 1949). Generalised Estimating Equation (GEE) regressions were fit to test the associations between thinking about quitting due to the tax increase and post-tax increases in quit intention, serious quit attempts, and quitting smoking (≤ 1 cigarette a month), as well as sociodemographic variables.

**Results:**

Circa half of the people who smoke reported thinking about quitting smoking due to the upcoming tax increase (Wave 1 = 51.3% (*n* = 1052); Wave 2 = 47.3% (*n* = 849)). Individuals who reported thinking about quitting smoking due to upcoming tax increase(s) were more likely to have increased their quit intention (aOR: 2.00, *p* ≤ .001) or have carried out a serious quit attempt (aOR:1.48, *p* ≤ .001) post-tax increase. More people attempted to quit smoking between wave 2 and 3 (post 2021 increase) than between wave 1 and 2 (post 2020 increase). We did not find an interaction effect between wave and thinking about quitting for quit intention, quit attempts, and quitting smoking.

**Conclusions:**

Tax increases stimulate people to think about quitting. Thinking about quitting due to an upcoming tax increase was associated with more positive quit intention and serious quit attempts.

## Background

Tobacco consumption remains a leading global cause of preventable morbidity and mortality [1]. Encouraging and helping people who smoke to quit is therefore a high priority for public health. Most people who smoke have expressed they want to quit smoking in the future [2]. According to the Transtheoretical Model (TTM) of Behaviour change, there are five stages of change in the process of change for smoking cessation: pre-contemplation, contemplation, preparation, action, and maintenance [3]. Most importantly for this paper, people who think about quitting are in the contemplation stage. This may be followed by having an intention to quit smoking (preparation stage), which may translate into carrying out a quit attempt (action stage). The majority of people who smoke are not successful at their first try to quit smoking. Estimates of average number of quit attempts needed to successfully quit smoking vary per study; but many practitioners believe it takes at least five attempts to successfully quit [4]. It is therefore important to repeatedly stimulate people who smoke to think about, consider and attempt to quit smoking.

Health concerns are generally considered the primary reason to want to quit smoking, followed by both social and financial concerns [5, 6]. More recent studies, in countries with high cigarette prices, have found that the cost of tobacco has become an increasingly important motive to quit smoking. A study in the United Kingdom found an increasing trend in cost as a motivation to quit, relative to other motivations, between 2018 and 2022 [7]. In Australia, which has the highest tobacco prices worldwide, the high cost of tobacco has even overtaken health reasons as the main motivation to change or attempt to change smoking behaviour since 2013 [8]. Between 2013 and 2020, the Australian government increased tobacco excise taxes annually by 12.5% [9]. The proportion of people who reported the high cost of tobacco as a motivation to change their smoking behaviour has increased from 38.1% (2007) to 56.7% (2019). A similar increase was found among successful quitters: cost was reported as a motivator to quit by 33.2% in 2007 and by 47.9% in 2019 [8]. Also in New Zealand a large increase in cost as a reason for quitting was found after a tax increase: more than twice as many people reported trying to quit smoking due to costs (25.5% versus 55.6%), and more people had carried out a quit attempt following the tax increase (30.0% versus 39.0%) [10]. A tax increase may work as a commitment device or trigger to make a quit attempt (e.g. “I will quit smoking before April 1, when taxes are increased”) [10, 11]. An individual using a tax increase as a commitment device may thus change their smoking behaviour out of their desire to quit, rather than out of — or in addition to, financial necessity [11].

In general, smoking prevalence is higher among people with a lower socioeconomic status (SES), that is, with a lower income and/or educational level, than people with a higher SES [12]. People with a lower SES are more likely to report the cost of tobacco as a reason to quit smoking than individuals with a higher SES [13, 14]. This social gradient is also visible in empirical econometric studies, which have found that lower SES individuals are more sensitive to price changes, and are more likely to reduce consumption or try to quit smoking as tobacco prices increase [15]. An opposite social gradient is found in quit success: individuals with a lower SES are less likely to successfully quit smoking than individuals with a higher SES [16, 17]. While high tobacco costs may be a reason to try to quit smoking, especially among lower SES individuals, this may not always translate into successful smoking cessation.

To stimulate tobacco cessation, increased tobacco taxation is one of the key measures of the tobacco control plan introduced by the government of the Netherlands in 2018. The tobacco control plan aims to achieve a Smokefree Generation in 2040, meaning less than 5% of adults smokes and 0% of children smoke. In 2017, prior to the new tobacco control plan, adult smoking prevalence in the Netherlands was 23.1% and prevalence was almost 10 percentage points higher among people with a low SES (25.7%) than with a high SES (16.8%) [18]. In April 2020, the first tax increase was implemented: an increase of €1 per pack of factory-made (FM) cigarettes and a €2.50 increase per pouch of roll-your-own (RYO) tobacco [18, 19]. On 1 January 2021, another small tax increase was implemented, entailing a minor rise of approximately 12 eurocents per pack of cigarettes and 30 eurocents per pouch of RYO tobacco [20]. This second tax increase was not part of the 2018 tobacco control plan, but a previously agreed upon increase. The tax increases were thus formally announced at least two years prior to implementation. Around the implementation date, the 2020 tax increase received a lot of national media attention. The smaller 2021 tax increase received much less media attention around the implementation date.

In this paper we examined whether people who smoke thought about quitting due to a forthcoming tax increase, and whether this is associated with cessation behaviours after the tax increase. Additionally, we analysed whether this association is similar across both the large (2020) and small (2021) tax increase, because the tax increases differed much in size. We also explored potential variations across age, education, and income (SES) subgroups in thinking about quitting due to the tax increase, because young adults and individuals with a low SES are key demographic groups for the Netherlands’ tobacco tax increase.

## Methods

### Sample

Longitudinal data from the International Tobacco Control (ITC) Policy Evaluation Netherlands Survey was used. The ITC Project is a prospective cohort study, which aims to assess the impact of the WHO FCTC policies. A nationally representative sample of smokers from the Netherlands was sampled from the TNS NIPObase, using quota on gender, age, and region. Respondents answered online surveys and were compensated for this with ‘NiPoints’, which can be used to acquire gift cards. At time of recruitment, respondents had to be at least 18 years old, have smoked at least 100 cigarettes in their life, and be at least a monthly smoker. Respondents did not have to be current smokers at follow-up. The sample is replenished with new respondents to compensate for people who were lost for follow up. Further details on sampling and the ITC methodology, as well as the full questionnaires, can be found elsewhere [21–23].

This study analyses data from Cohort 2 of the ITC Netherlands surveys. Fieldwork was conducted at an interval of seven months: February–March 2020 (Wave 1), September–November 2020 (Wave 2), and June-July 2021 (Wave 3). The large tax increase (April 2020) was implemented between Wave 1 and Wave 2, and the small tax increase (January 2021) between Wave 2 and Wave 3. The response rate of the ITC Netherlands Surveys was between 54.0% and 58.3% (Wave 1: 57.6%; Wave 2: 58.3%; Wave 3: 54.0%); retention between surveys was 82.0% (Wave 2) and 83.0% (Wave 3) [21, 24, 25]. In total, our sample consisted of 5919 observations (Wave 1: *n* = 2051; Wave 2: *n* = 1919; Wave 3: *n* = 1949) by 2734 respondents.

### Measures

#### Outcome measures

Quit intention, quit attempts, and successful quitting were our post-tax outcome measures. Quit intention was measured by asking current smokers whether they were planning to quit smoking (within the next month; between 1–6 months from now; sometime in the future, beyond 6 months; not planning to quit; don’t know). Respondents who stated having an intention to quit within the next month or between 1–6 months were coded as having an intention to quit within 6 months [1] versus those wanted to quit in the future beyond 6 months, were not planning to quit or did not know (0). Quit attempts were defined as having made at least one serious quit attempt since the last survey date. Respondents were coded as having made a serious quit attempt (1) or not having made a serious quit attempt, or not knowing (0). Among those who reported to have attempted to quit smoking, respondents were classified as successful quitters if they indicated at the time of completing the survey to have quit smoking or smoked less than one cigarette a month. Respondents were classified as unsuccessful quitters if they indicated to have made a serious a serious quit attempt but smoked again at the time of completing the survey.

#### Thinking about quitting due to the upcoming tax increase

Prior to each tax increase, respondents were asked: “The tax on cigarettes and rolling tobacco will increase in [April 2020/January 2021]. Will this increase in tax make you think about quitting smoking?”. The question did not include how much the taxes would increase. Responses were categorised into two groups: tax encouraged to think about quitting (1—somewhat; a lot), and tax did not encourage to think about quitting (0—not at all; don’t know).

#### Covariates

All models were adjusted for the following sociodemographic variables: gender (man; woman), age group (18–24; 25–39; 40–54; 55 and over), educational level (low; moderate; high), income level (low; moderate; high; not stated), and region (North, East, South, West). Level of education was coded as low (primary education and lower pre- vocational secondary education), moderate (middle prevocational secondary education and secondary vocational education), or high (senior general secondary education, pre- university education, and higher professional education). Monthly gross household income was categorised as low (< €2000), moderate (€2000–3000), high (> €3000), and not stated (don’t want to say or don’t know). Models set up to explore post-tax increase behaviours were also adjusted for three pre-tax increase smoking characteristics: nicotine dependence, type of tobacco smoked, and having carried out a previous quit attempt. Nicotine dependence was measured using the Heaviness of Smoking Index (HSI), a six-point scale that is the sum of two categorised measures: number of cigarettes per day and time to first cigarette after waking up [26]. A higher HSI score indicates a higher nicotine dependence. Type of tobacco refers to what tobacco the individual reported to use (factory-made cigarettes; roll-your-own tobacco; both [dual use]). Having carried out a previous serious quit attempt was added as a dummy variable (having carried out a previous serious quit attempt; or not). Finally, a variable that adjusted for the self-reported effect of the SARS-COV-19 pandemic on smoking behaviour was included (“What effect has the coronavirus outbreak had on your smoking?”) and categorised as: reporting to smoking less or quitting (because of it I quit smoking; because of it I smoke less), reporting no effect (it had no effect at all on my smoking; don’t know), or reporting to smoke more (because of it I’m smoking more). The first cases of the SARS-COV-19 virus in the Netherlands were reported during the fieldwork of Wave 1; items about the effect of the outbreak on smoking behaviour were thus only included in Wave 2 and Wave 3.

### Statistical analyses

All analyses were carried out in SPSS 29. Data were weighted using rescaled weights to represent the Dutch population of people who smoke according to Statistics Netherlands at Wave 1, 2 and 3, by sex and age, educational level, and region [21]. First, a Generalised Estimating Equation (GEE) regression was fit to examine the association between thinking about quitting due to the tax increase and sociodemographic variables. Interactions between wave and gender, age, education, and income were also tested to check whether the relations with sociodemographic variables were stable across waves. Another set of GEEs was fit to examine whether reporting before the tax increases that the upcoming tax increase made them think about quitting was associated with cessation behaviours after the tax increases. Quit intention, quit attempts, and smoking cessation at Wave 2 (post major tax increase) and Wave 3 (post minor tax increase) were the outcome measures. Models were adjusted for sociodemographic variables (gender, age group, income level, education level, region), smoking behaviour at the previous wave (type of tobacco smoked, HSI, and previous quit attempts), and self-reported effect of the coronavirus outbreak on smoking behaviour. Interactions between thinking about quitting due to price and Wave were modelled in separate analyses. All GEEs were fit with binomial distributions, logit link, and the unstructured correlation structure.

Multiple imputation was employed to fill in missing values on predictor variables to increase the sample size and to accommodate for possible bias in the analysis. Although outcome variables were also involved in the imputation phase, imputed values on these variables were not used in the analysis phase, because excluding cases with missing values on the outcome variable yields more stable estimates [27]. The percentage of missing values in the original data used in the multiple imputations for the analysis on the relation between thinking about quitting due to a forthcoming tax increase was 20.4%, whereas the percentage of missing values in the original data used in the multiple imputation for the other research questions was 22.8%. The number of imputations was set at 100 per analysis; pooled estimates are reported. Missing data were imputed, using the full conditional specification method (with the sequential regression procedure) [27]. Simulation studies indicated that this imputation method produces unbiased parameter estimates and standard errors [28, 29]. By taking the number of imputations at least as large as the percentage of incomplete cases, in the present analysis 100 imputations, this is estimated to yield an acceptable power loss and acceptable uncertainty on the *p*-value [30]. All variables in the GEE models were used as predictors for the multiple imputations. In both the imputation and analysis phase, care was taken of possible clustering effects due to measurements being nested within persons.

## Results

Focusing on the largest subgroups, of the respondents in this study (*n* = 2734) 54.5% were men, 30.4% were over 55 years old, 32.5% had a moderate income, 40.5% had a moderate educational level, and 46.5% lived in the west of the Netherlands (Table 1). At each Wave, the majority smoked factory-made cigarettes, did not carry out a serious quit attempt in the last six months, and did not change their smoking behaviour due to the SARS-COVID-19 outbreak. Average nicotine dependence was comparable across the three waves. In Wave 1, prior to the major tax increase, 51.3% (*n* = 1052) of respondents who smoked indicated that they thought about quitting due to the upcoming tax increase, while 48.7% (*n* = 999) did not. In Wave 2, prior to the minor tax increase, 47.3% (*n* = 849) of the respondents who smoked indicated they thought about quitting due to the upcoming tax increase, while 52.7% (*n* = 946) did not.

**Table 1:** Participant characteristics overall﻿ (unique respondents), and per wave

	**Overall** (*N* = 2734)	**Wave 1** (*n* = 2051)	**Wave 2** (*n* = 1919)	**Wave 3** (*n* = 1949)
	N (%)	N (%)	N (%)	N (%)
Gender				
Woman	1245 (45.5)	905 (44.1)	848 (44.2)	862 (44.2)
Man	1489 (54.5)	1146 (55.9)	1071 (55.8)	1087 (55.8)
Age				
18–24 years	432 (15.8)	290 (14.2)	279 (14.5)	261 (13.4)
25–39 years	720 (26.3)	579 (28.2)	526 (27.4)	492 (25.2)
40–54 years	750 (27.4)	557 (27.1)	519 (27.1)	564 (28.9)
55 + years	832 (30.4)	625 (30.5)	595 (31.0)	632 (32.5)
Income				
Low	869 (31.8)	650 (31.7)	606 (31.6)	594 (30.5)
Moderate	889 (32.5)	678 (33.0)	640 (33.4)	664 (34.1)
High	353 (12.9)	246 (12.0)	244 (12.7)	261 (13.4)
Not stated	577 (21.1)	433 (21.1)	429 (22.3)	429 (22.0)
Education				
Low	1051 (38.7)	799 (39.3)	717 (37.6)	715 (36.9)
Moderate	1098 (40.5)	824 (40.5)	776 (40.7)	782 (40.3)
High	565 (20.8)	412 (20.1)	413 (21.7)	442 (22.8)
Region				
West	1271 (46.5)	953 (46.5)	899 (46.9)	910 (46.7)
North	325 (11.9)	239 (11.6)	230 (12.0)	231 (11.9)
East	561 (20.5)	426 (20.8)	384 (200)	405 (20.8)
South	577 (21.1)	433 (21.1)	406 (21.2)	402 (20.6)
Smoking status				
Smoker		2051 (100)	1796 (93.6)	1766 (90.6)
Quitter		0 (0)	123 (6.4)	183 (9.4)
Thought about quitting due to upcoming tax^a^				
Yes		1052 (51.3)	849 (47.3)	-
No		999 (48.7)	946 (52.7)	-
Type of tobacco^a^				
FM cigarettes		1181 (57.7)	1062 (59.3)	1006 (57.2)
RYO tobacco		471 (23.0)	438 (24.5)	466 (26.5)
Both		396 (19.3)	291 (16.2)	286 (16.3)
HSI (mean (SD))^a^		2.25 (1.55)	2.27 (1.54)	2.24 (1.55)
Quit attempt				
None		1344 (66.5)	1433 (76.2)	1370 (71.2)
At least one		678 (33.5)	449 (23.8)	554 (28.8)
COVID				
Smoke more		-	227 (11.8)	199 (10.3)
No effect on smoking		-	1371 (71.4)	1412 (72.9)
Smoke less or quit		-	321 (16.8)	326 (16.8)

*FM* Factory-made cigarettes, *RYO* tobacco Roll-your-own tobacco, *HSI* Heaviness of Smoking Index, Quit attempt conducted at least one serious quit attempt in the last six months or since last survey date, *COVID* self-reported effect of the coronavirus outbreak on smoking behaviour

^a^ Among people who smoke

Table 2 displays the associations between sociodemographic variables and thinking about quitting smoking. Women (versus men), 18–24 year olds (versus 40–54 and 55 and over), individuals with a low income (versus not stated), and those living in the west of the Netherlands (versus the south) were more likely to report that the tax increase made them think about quitting. An interaction between education and wave was found. The ratio of the odds of reporting to think about quitting for individuals with a high or moderate educational level versus individuals with a low educational level was larger in Wave 2 than in Wave 1 (ratio of aORs: 1.37, *p* = 0.039 and 1.34, *p* = 0.014, respectively). To explore this interaction, Table 2 also displays for each wave the aORs of low versus moderate, and of low versus high education. In Wave 1 low educated persons were less likely to report that tax increase made them think about quitting compared to moderately educated persons, and in wave 2 this was also the case when compared to highly educated persons. The aORs of wave 2 versus wave 1 for each educational level in Table 2 show that individuals with a lower educational level were significantly less likely to think about quitting in Wave 2 than in Wave 1, but that there was no relation between wave and think about quitting for individuals with a moderate or high educational level. No other significant interactions were found (not displayed).

**Table 2:** Associations with thinking about quitting due to the tax increase

	**Tax increase made you think about quitting**
	aOR (95% CI)
Gender	
Woman	1
Man	0.85 (0.73 – 0.99)*
Age	
18–24 years	1
25–39 years	0.85 (0.67—1.07)
40–54 years	0.70 (0.55—0.89)**
55 + years	0.52 (0.41—0.66)***
Income	
Low	1
Moderate	1.03 (0.85—1.24)
High	0.85 (0.66—1.10)
Not stated	0.79 (0.64 – 0.97)*
Region	
West	1
North	0.98 (0.76—1.26)
East	0.87 (0.71 – 1.07)
South	0.74 (0.62—0.90)**
Education*Wave 1	
Low	1
Moderate	1.24 (1.01 – 1.51)*
High	1.18 (0.91 – 1.54)
Education*Wave 2	
Low	1
Moderate	1.57 (1.27 – 1.95)***
High	1.48 (1.13 – 1.94)**
Wave*low education (*additional exploration)*	
Wave 1	1
Wave 2	0.74 (0.62–0.87) ***
Wave*moderate education (*additional exploration)*	
Wave 1	1
Wave 2	0.93 (0.81–1.08)
Wave*high education (*additional exploration)*	
Wave 1	1
Wave 2	0.92 (0.74–1.14)

^*^p ≤ .05, ** *p* ≤ .01, *** *p* ≤ .001

Table 3 displays the associations between thinking about quitting due to the upcoming tax and post-tax increase cessation behaviours that follow from the GEE analyses. Individuals who reported thinking about quitting smoking due to the upcoming tax increase(s) were more likely to report an intention to quit smoking (aOR: 2.00, *p* ≤ 0.001), to have carried out at least one quit attempt (aOR:1.48, *p* ≤ 0.001), but not to have successfully quit smoking (aOR:0.95, *p* = 0.785) than individuals who reported to not think about quitting due to the tax increase(s). No significant interactions between thinking about quitting due to tax increases and wave were found (quit intention: aOR:0.80, *p* = 0.189; quit attempts: aOR:0.98, *p* = 0.920; quit success: aOR:1.58, *p* = 0.236) (not displayed). Individuals were more likely to carry out quit attempts in wave 3 than in wave 2 (aOR:1.80, *p* ≤ 0.001).

**Table 3:** Associations with post-tax increase cessation behaviours

	**Quit intention**	**Quit attempts**	**Quit smoking**
	aOR (95% CI)	aOR (95% CI)	aOR (95% CI)
Think about quitting due to tax^a^			
No	1	1	1
Yes	2.00 (1.64—2.45)***	1.48 (1.22 – 1.79)***	0.95 (0.65 – 1.39)
Wave			
2	1	1	1
3	0.88 (0.75—1.03)	1.80 (1.45 – 2.24)***	0.86 (0.58 – 1.26)
Gender			
Woman	1	1	1
Man	0.93 (0.75—1.14)	0.84 (0.70 – 1.00)	1.17 (0.81 – 1.70)
Age			
18–24 years	1	1	1
25–39 years	1.33 (0.96—1.85)	0.99 (0.74 – 1.33)	1.07 (0.61 – 1.88)
40–54 years	1.26 (0.89 – 1.79)	0.96 (0.70 – 1.31)	0.99 (0.55 – 1.79)
55 + years	0.97 (0.68 – 1.39)	0.90 (0.65 – 1.23)	0.86 (0.47 – 1.57)
Income			
Low	1	1	1
Moderate	0.92 (0.72—1.18)	0.85 (0.68 – 1.05)	1.35 (0.86 – 2.11)
High	0.82 (0.59 – 1.15)	0.69 (0.50 – 0.94)*	1.92 (1.05 – 3.50)*
Not stated	0.69 (0.52—0.91)**	0.82 (0.64 – 1.04)	1.43 (0.88 – 2.32)
Education			
Low	1	1	1
Moderate	1.41 (1.11—1.79)**	1.08 (0.87 – 1.33)	1.00 (0.65 – 1.56)
High	1.72 (1.27 – 2.32)***	1.11 (0.84 – 1.45)	1.00 (0.59 – 1.69)
Region			
West	1	1	1
North	0.93 (0.67—1.30)	0.73 (0.55 – 0.98)*	0.35 (0.15 – 0.82)*
East	0.90 (0.69—1.17)	0.87 (0.68 – 1.09)	1.22 (0.76 – 1.95)
South	0.88 (0.68 – 1.13)	1.02 (0.81 – 1.27)	1.62 (1.06 – 2.48)*
Type of tobacco^a^			
FM cigarettes	1	1	1
RYO tobacco	0.73 (0.54 – 0.99)*	1.10 (0.85—1.43)	0.67 (0.40—1.14)
Both	0.77 (0.58—1.01)	1.01 (0.78—1.30)	0.85 (0.50 – 1.44)
HSI (0–6)^a^	0.99 (0.92—1.07)	1.02 (0.95—1.09)	0.89 (0.77 – 1.02)
Quit attempt^a^			
None	1	1	1
At least one	2.74 (2.22—3.37)***	5.82 (4.46 – 7.60)***	0.58 (0.40 – 0.86)**
COVID			
Smoke more	1	1	1
No effect on smoking	0.76 (0.60 – 0.95)**	0.85 (0.68 – 1.23)	6.58 (2.76 – 15.68)***
Smoke less or quit	1.99 (1.44 – 2.74)***	2.82 (2.04 – 3.91)***	11.99 (4.80 – 29.94)***

*FM* Factory-made cigarettes, *RYO tobacco* Roll-your-own tobacco, *HSI* Heaviness of Smoking Index, Quit attempt conducted at least one serious quit attempt in the last six months or since last survey date, *COVID*  self-reported effect of the coronavirus outbreak on smoking behaviour

^a^Measured at t-1

*p ≤ .05, ** *p* ≤ .01, *** *p* ≤ .001

## Discussion

This study explored whether people who smoke thought the forthcoming tax increase(s) would make them think about quitting, and whether this was associated with cessation behaviour post-tax increase. We found that approximately half of the people who smoke reported thinking about quitting smoking due to an upcoming tax increase. People were more likely to report thinking about quitting due to the tax increase before the 2020 tax increase, than before the 2021 tax increase. This was an expected outcome, because the 2020 tax increase was a much larger tax increase than in 2021. Additionally, the 2020 tax increase received a lot of media attention prior to the increase, while the 2021 tax increase did not.

We found that reporting to think about quitting due to the upcoming tax increase was positively associated with having a quit intention and having carried out at least one serious quit attempt post-tax increase. Our results are similar to a study conducted in Minnesota, USA, following a large tax increase, which also found a positive association between reporting to think about quitting due to a tax increase and cessation behaviours at follow up [11]. We did not find an association between thinking about quitting due to the tax increase and successful quitting. This was in line with a study by Keeler et al., which found positive associations between cigarette price and both quit intention and quit attempts, but not with successful smoking cessation among White Americans [31]. It thus appears that an upcoming tax increase encourages and motivates people to think about or attempt quitting, but more is sometimes needed for it to translate into successful smoking cessation. Combining tax increases with other tobacco control measures, such as providing cessation support, may help in making quit attempts successful.

Regarding differences in cessation behaviours between the small and the large tax increase, we found that people were more likely to carry out a quit attempt in Wave 3, after the minor tax increase in 2021, than in Wave 2, after the major tax increase in 2020. An explanation may be that this is the cumulative effect of multiple tax increases. Repeated tax increases could provide people with repeated cues to think about quitting, which may stimulate people to move towards cessation. Another explanation may be a seasonal effect, as tobacco consumption is highest in summer [32], and more people attempt to quit smoking in January due to New Year’s resolutions [33]. No interactions between wave and thinking about quitting due to the upcoming tax increases in relation to the cessation behaviours were found. However, it appears that there is a greater impact of a larger tax increase through the mediating effect of thinking about quitting. This is the case for lower-educated individuals. Lower-educated individuals are more likely to think about quitting due to the large (Wave 1) than due to the small tax increase (Wave 2) (see Table 2) and being more likely to think about quitting in turn is associated with being more likely to carry out a quit attempt or having an intention to quit (see Table 3).”

Another aim of this study was to explore potential variations across age, education, and income subgroups. Our finding that 18–24 year olds were generally more likely to think about quitting due to the upcoming tax increase than people aged over 40 is in line with the literature which posits that younger people are more sensitive to price changes than older people [15, 34]. There were two unexpected findings. First, people with a low income were more likely to think about quitting due to the upcoming tax increase than people who did not state their income; no differences were found between people with a low and high income. Second, we found that people with a lower educational level were less likely to report to think about quitting due to the upcoming tax increase than people with a moderate and high educational level prior the small tax increase (Wave 2), and less likely than people with a moderate educational level prior the large tax increase (Wave 1). Both these findings contrast the literature which posits that individuals with a lower SES (income and/or education) are more responsive to price [35–37], or report cost as a reason to quit smoking [11, 14], than higher SES individuals. An explanation may be that we specifically asked about the upcoming tax increase, rather than the actual price of cigarettes or cost related to smoking.

Several limitations need to be kept in mind when interpreting our results. First of all, there are limitations related to using self-reported data from a questionnaire. There may be social desirability bias in reporting whether someone will think about quitting due to the tax increase. People might have reported thinking about quitting smoking due to the tax increase because they feel they are expected to think about quitting rather than actually considering to quit. The risk of social desirability bias was mitigated by the anonymous nature of the ITC Surveys: the questionnaires are completed via the internet, and respondents are reminded of anonymity prior to consenting to each ITC questionnaire. Additionally, we did not specify how much the taxes would increase in the questionnaire. It may thus have been that the respondents were not aware of the size of each tax increase. However, the large tax increase received substantial, national media attention around the implementation making it unlikely for someone who smokes to have not known. The small tax increase also received media attention, but was less highly publicised. Furthermore, our findings are restricted to the situation at the time of data collection: successfully having quit smoking was operationalised as having quit at least one month at the time of completing the survey. Therefore, we could have overlooked people who relapsed prior to our survey or included people who have quit smoking within one month before the survey. Finally, not all measures in this manuscript are validated measures, since, to the best of our knowledge, there is no validated measure for thinking about quitting due to upcoming tax increase(s). It could be that our measure is more related with having a quit intention, than considering to quit due to the upcoming tax increase. Further research to validate the measure would be recommended.

## Conclusions

Tobacco tax increases stimulate people to think about quitting. About half of the people who smoke reported they thought about quitting due to the upcoming tax increase, and the people who reported this were more likely to have a quit intention and made a serious quit attempt post-tax increase compared with people who did not think about quitting due to the tax increase(s). There was no relation with successful quitting. Tax increases could serve as a trigger for people to evaluate their smoking behaviour, as well as serve as an immediate incentive to try to quit smoking.

## Data Availability

In each country participating in the international Tobacco Control Policy Evaluation (ITC) Project, the data are jointly owned by the lead researcher(s) in that country and the ITC Project at the University of Waterloo. Data from the ITC Project are available to approved researchers 2 years after the date of issuance of cleaned data sets by the ITC Data Management Centre. Researchers interested in using ITC data are required to apply for approval by submitting an International Tobacco Control Data Repository (ITCDR) request application and subsequently to sign an ITCDR Data Usage Agreement. The criteria for data usage approval and the contents of the Data Usage Agreement are described online (http://www.itcproject.org).

## Abbreviations

ITC: International Tobacco Control Policy Evaluation Project
TTM: Transtheoretical Model
SES: Socioeconomic status
FM: Factory-made (cigarettes)
RYO: Roll-your-own (tobacco)
WHO: World Health Organization
FCTC: Framework Convention on Tobacco Control
HIS: Heaviness of Smoking Index
AOR: Adjusted Odds Ratio
SARS-COV-19: Coronavirus Disease 2019
GEE: Generalised Estimating Equation
6m: Six months
CI: Confidence interval
